# A diVIsive Shuffling Approach (VIStA) for gene expression analysis to identify subtypes in Chronic Obstructive Pulmonary Disease

**DOI:** 10.1186/1752-0509-8-S2-S8

**Published:** 2014-03-13

**Authors:** Jörg Menche, Amitabh Sharma, Michael H Cho, Ruth J Mayer, Stephen I Rennard, Bartolome Celli, Bruce E Miller, Nick Locantore, Ruth Tal-Singer, Soumitra Ghosh, Chris Larminie, Glyn Bradley, John H Riley, Alvar Agusti, Edwin K Silverman, Albert-László Barabási

**Affiliations:** 1 Center for Complex Networks Research and Department of Physics, Northeastern University, Boston, MA, 02115, USA; 2 Center for Cancer Systems Biology, Dana-Farber Cancer Institute, Boston, MA, 02215, USA; 3 Department of Theoretical Physics, Budapest University of Technology and Economics, Budapest, 1111, Hungary; 4 Channing Division of Network Medicine and Division of Pulmonary and Critical Care Medicine, Department of Medicine, Brigham and Women's Hospital, Harvard Medical School, Boston, MA, 02115, USA; 5 GlaxoSmithKline, King of Prussia, PA, 19406, USA; 6 University of Nebraska Medical Center, Omaha, NE, 68198, USA; 7 Pulmonary and Critical Care Division, Brigham and Women's Hospital, Harvard Medical School, Boston, MA, 02115, USA; 8 GlaxoSmithKline, Research Triangle Park, NC, 27709, USA; 9 GlaxoSmithKline, Stevenage, Hertfordshire, SG1 2NY, UK; 10 Hospital Clinic, IDIBAPS, Thorax Institute, University of Barcelona, 08036, Barcelona; 11 Center for Network Science, Central European University, Budapest, 1051, Hungary; 12 Department of Medicine, Brigham and Women's Hospital, Harvard Medical School, Boston, MA, 02115, USA; 13 FISIB, CIBERES, Mallorca, 07110, Spain

**Keywords:** Chronic Bronchitis, COPD, Emphysema, subtyping, gene expression analysis

## Abstract

**Background:**

An important step toward understanding the biological mechanisms underlying a complex disease is a refined understanding of its clinical heterogeneity. Relating clinical and molecular differences may allow us to define more specific subtypes of patients that respond differently to therapeutic interventions.

**Results:**

We developed a novel unbiased method called diVIsive Shuffling Approach (VIStA) that identifies subgroups of patients by maximizing the difference in their gene expression patterns. We tested our algorithm on 140 subjects with Chronic Obstructive Pulmonary Disease (COPD) and found four distinct, biologically and clinically meaningful combinations of clinical characteristics that are associated with large gene expression differences. The dominant characteristic in these combinations was the severity of airflow limitation. Other frequently identified measures included emphysema, fibrinogen levels, phlegm, BMI and age. A pathway analysis of the differentially expressed genes in the identified subtypes suggests that VIStA is capable of capturing specific molecular signatures within in each group.

**Conclusions:**

The introduced methodology allowed us to identify combinations of clinical characteristics that correspond to clear gene expression differences. The resulting subtypes for COPD contribute to a better understanding of its heterogeneity.

## Background

Chronic obstructive pulmonary disease (COPD) is one of the most prevalent chronic diseases (4th cause of death globally), with increasing incidence worldwide. Understanding of the disease pathobiology is far from complete and only few novel therapeutic mechanisms of action have been identified. Tobacco smoking is the main risk factor for COPD, but only a fraction of all smokers develops the disease [1]. This variable response to smoking, plus the observation that COPD aggregates in families, strongly suggest a genetic component to the disease [2-6]. Yet, COPD is a very heterogeneous and complex disease, with varied pulmonary and extra-pulmonary clinical manifestations [7]. Understanding and characterizing this biological and clinical heterogeneity could help identify subgroups of patients (subtypes) that may benefit from different therapeutic strategies [8]. To investigate the genomic and pathobiological basis of COPD subtypes with distinct clinical manifestations, we applied several novel and complementary computational strategies to differential gene expression analysis. We used expression data from induced sputum samples of former smokers with COPD and varying degree of airflow limitation. The patients are a subset of the large ECLIPSE cohort, which is a multi-center, 3 year observational international study that collected clinical, genetic, proteomic and biomarker measures in a population of COPD patients [9]. Specifically, in the current study we sought to: (*i *) compare the gene expression pattern between patient groups with different clinical characteristics; (*ii*) conversely, assess the clinical characteristics of groups of patients with distinct gene expression patterns identified by a novel diVIsive Shuffling Approach (VIStA) developed specifically for this purpose (see below). Unexpectedly, we found that the reverse approach (*ii*) showed greater potential to identify specific pathways that may offer novel therapeutic targets [10] than the traditional approach (*i*).

## Methods

### Study design, participants and ethics

The ECLIPSE cohort is a large, prospective, observational and controlled study (Clinicaltrials.gov identifier NCT00292552; GSK study code SCO104960), whose design has been published previously [9]. Here, we investigated differential gene expression in induced sputum samples of a subset of the participants that included 140 former smokers with COPD (70 with moderate or GOLD stage 2 and 70 with severe or GOLD stage 3-4 airflow limitation, matched for age and gender) with characterized clinical and laboratory measures (Table 1). Sputum induction and processing with dithiothreitol (DTT) was performed using standard methods as described previously [5], details on the generation and processing of the expression data can be found in [3]. The ECLIPSE study complies with the Declaration of Helsinki and Good Clinical Practice Guidelines and was approved by the Ethics Committees and Institutional Review Boards of all participating centers. All participants provided written informed consent.

**Table 1:** Summary of the characteristics of 140 subjects with sputum gene expression data from the ECLIPSE Cohort.

Demographics and clinical data	
Age, yrs.	65 ± 5.5
Males, %	66
Body mass index, Kg/m^2^	26.8 ± 5.2
Smoking exposure, pack-yrs.	48.3 ± 29.1
Annual Exacerbation rate, year^-1^	0.98 ± 1.6

**Lung function**	

FEV1, L	1.26 ± 0.45
FEV1, % revers.	9.5 ± 10.4
FEV1/FVC, %	43.2 ± 11.5

**Imaging**	

Emphysema, -950HU %	19.2 ± 12.2
Emphysema, extent code	2.8 ± 1.8

**Systemic inflammation**	

hsCRP (mg/L)	8.24 ± 15.0
IL6 (pg/mL)	7.8 ± 36
IL8 (pg/mL)	9.3 ± 5.2
CCL18 (ng/mL)	121.7 ± 46
Fibronogen (mg/dL) **-**	481.9 ± 107.6
TNFA (ng/mL)	103.2 ± 624
SPD (ng/mL)	120.6 ± 78

**Induced sputum**	

Total cell count, × 10^6^	7.5 ± 1.78
Neutrophils, %	64.8 ± 8.5
Eosinophils, %	3.1 ± 2.04
Lymphocytes, %	25.4 ± 7.9

Note that all subjects are COPD patients and former smokers. The values represent mean *± *standard deviation, frequency or proportion, as appropriate.

### Selection of clinical measures

Table 2 shows the clinical measures selected by COPD experts (SR, BC, AA, EKS) based on their association to important clinical outcomes (e.g. exacerbations, hospitalizations and death). The degree of airflow limitation (GOLDCD) was determined using spirometry, distances walked over 6 minutes (DWALK) were measured using standard methodology. Standardized questionnaires were used to obtain smoking status, cough and sputum (PHLEGM) production. COPD exacerbations in the year prior to the study were recorded, as well as body mass index (BMI). All subjects underwent a low-dose computed tomography (CT) scan of the chest to determine both airway disease and emphysema (FV950 as a quantitative assessment, and EMPHETCD as a radiologists score) [11]. Several inflammatory biomarkers were measured in peripheral blood [12]. For details on the definitions and acquisition procedures of the above measures see [9].

**Table 2:** Summary of the clinical characteristics of COPD patients identified as most relevant by clinical experts.

Category	Continuous Variable for Quantitative Analysis	Discrete Variable	Bins	Characteristics	Differentially expressed genes at FDR < 0.05
Chronic Bronchitis	Not applicable	Cough with Phlegm for at least 3 mos/yr for at least 2 years	low extreme (Q1 = 64)	neither chronic cough nor chronic phlegm	0
	
			high extreme (Q4 = 46)	both chronic cough and chronic phlegm	

History of Exacerbations	Number of exacerbations per year	2 or more per year and less than 2 per year	low extreme (Q1 = 26)	0 - Never	0
	
			high extreme (Q4 = 17)	3 - Always	

Body Mass Index (Kg/m^2^)	BMI	BMI < 21, 21-30, > 30	low extreme (Q1 = 18)	BMI < 21	0
	
			high extreme (Q4 = 35)	BMI > 30	

Airflow Limitation severity	FEV1 (% predicted)	GOLD Stage	low extreme (Q1 = 69)	< 2-GOLD stage	6,049
	
			high extreme (Q4 = 13)	>4 GOLD stage	

6 Minute Walk Distance	Quantitative 6MWD	< 350 meters and > 350 meters	low extreme (Q1 = 38)	>350 meters	0
	
			high extreme (Q4 = 101)	>350 meters	

Radiologist Emphysema	Emphysema severity category:		low extreme (Q1 = 40)	0-1.5 -No emphysema	0
				
assessment	Not affected (N): 0	Yes/No/Uncertain	high extreme (Q4 = 45)	4-5 - severe	
				
	Trivial (T): 1				
				
	Mild (M) 5-25%: 2				
				
	Moderate (O) 25-50%: 3				
				
	Severe (S) 50-75%: 4				
				
	Very Severe (V) > 75%: 5				

Densitometric Emphysema	Emphysema at -950 HU	Emphysema >10% (Yes/No)	low extreme (Q1 = 37)	Emphysema >10% = No	0
	
			high extreme (Q4 = 95)	Emphysema >10% = yes	

CT Airway Disease	Pi10 (Square root of wall area of 10 mm internal perimeter airways)	GOLD Stages 2-4 with Emphysema < 5% (Yes) or > 5% (No)	low extreme (Q1 = 63)	Trivial (< %5)	0
	
			high extreme (Q4 = 33)	Severe (50-75%, very severe (>75%))	

Columns 4-6 show the results of the differential gene expression analysis comparing the subjects of the defined bins or extremes for each characteristic. Q1/Q4 refer to the number of patients in the respective group (1st and 4th "quartile"). The only single characteristic yielding significantly differentially expressed genes is the degree of airflow limitation as given by GOLD stage (GOLDCD).

Note that there were no controls with normal lung function among the subjects. Hence, we cannot compare COPD to normal but only the differences between COPD patients [1].

### Relationship between clinical characteristics and gene expression

To investigate the relationships between differences in gene expression and clinical trait occurrence, we used two complementary analyses:

(i) For each of the clinical characteristics introduced above, we divided the patients into two groups based on clinically relevant cut-points (Table 2, column 5) and computed gene expression differences between the two groups. Gene expression analysis was performed using Significance Analysis of Microarrays (SAM) [13] with a false discovery rate (FDR) of 5% as cutoff.

(ii) We used VIStA (see below) to identify groups of patients with maximized differential gene expression and then compared their clinical characteristics.

### diVIsive Shuffling Approach (VIStA)

**Figure 1:**
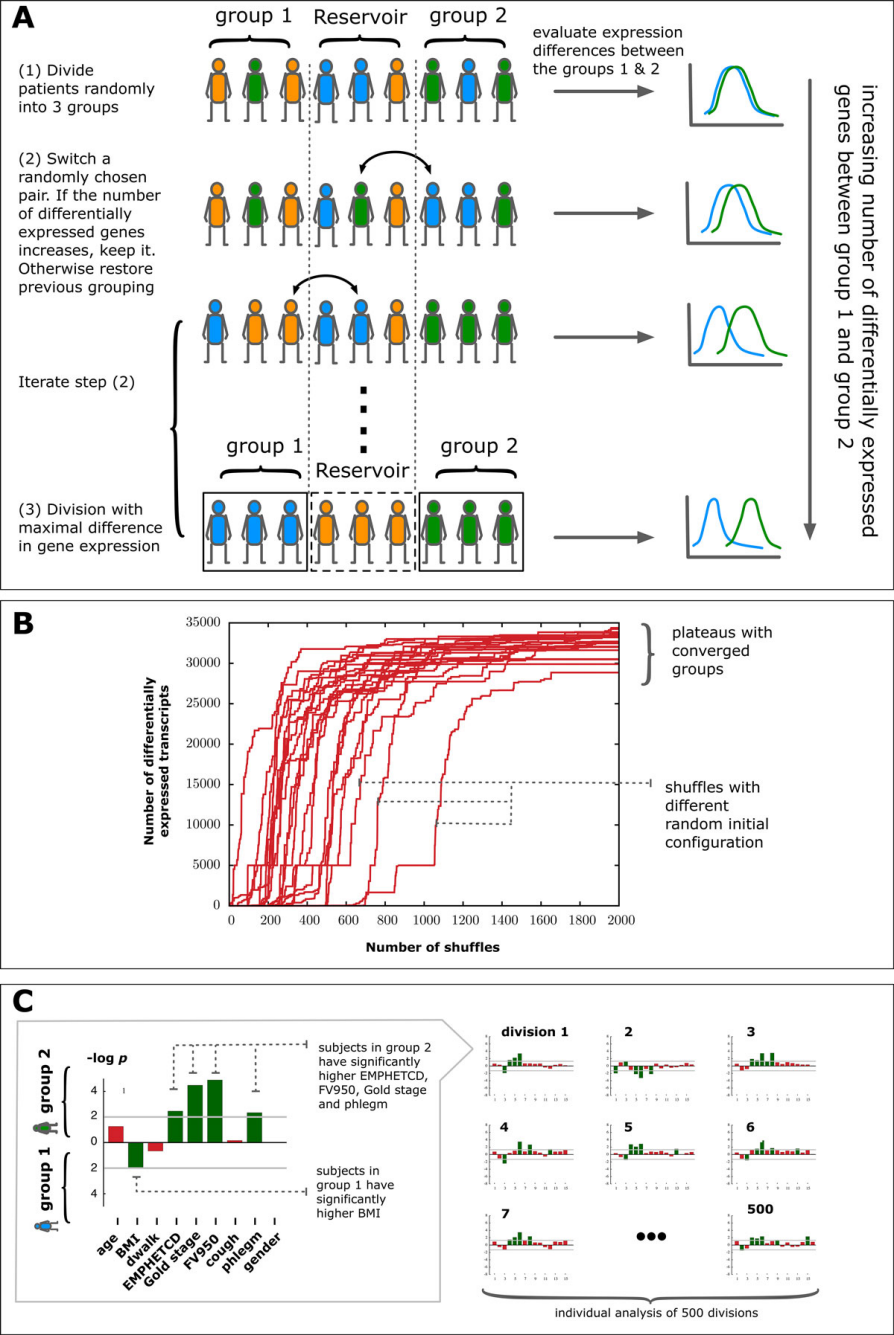
**Schematic representation of the diVIsive Shuffling Approach (VIStA)**. **A **Initially the subjects are divided randomly into three groups; gene expression differences are calculated between group 1 & 2, the third group serves as a reservoir for the subsequent shuffling steps. At each shuffling step, a subject from group 1 or 2 is randomly exchanged with a subject from the reservoir. If the number of differentially expressed genes increases thereby, the swap is accepted, otherwise rejected. **B **20 exemplary time series of the number of differentially expressed genes between group 1 & 2 as a function of the number of attempted shuffles. The different curves correspond to different random initial divisions. After approximately 1000 shuffles the groups converge and present a large, stationary number of differentially expressed genes. **C **For each of the obtained divisions (500 in total), clinical characteristics in group 1 & 2 are compared.

We developed a novel unbiased method called diVIsive Shuffling Approach (VIStA) to identify groups of patients with maximal difference in gene expression. The algorithm consists of the following steps:

(iii) *n *subjects are randomly partitioned into three groups of comparable size (Figure 1A). A SAM analysis is performed and the number of genes differentially expressed between groups 1 and 2 is counted. Group 3 serves as a "reservoir" of individuals for the subsequent steps.

(iv) An individual from group 1 or 2 is randomly swapped with an individual from the reservoir group 3. We repeat the SAM analysis, counting again the new number of differentially expressed genes (Figure 1A). If this count increases, the swap is accepted, otherwise rejected.

(v) Step (*ii*) is iterated until the number of differentially expressed genes reaches a plateau (Figure 1B), typically after approximately 1000 attempted swaps. The corresponding groups 1 & 2 represent a combination of patients with high differential gene expression.

Starting with different random initial configurations, we repeat the whole procedure (*i*) through (*iii*) 500 times, resulting in 500 end configurations, each characterized by a large number of differentially expressed genes. In order to explore the extent to which these 500 subdivisions are clinically relevant and distinct, we analyze them individually for statistically significant differences in clinical characteristics between the members of group 1 and 2. For each subdivision, we identify the set of clinical characteristics (Table 2) that differ significantly between patients in group 1 and group 2 using a Mann-Whitney-U-test (significance threshold of *p-*value *≤ *0.05) for all continuous characteristics (e.g. BMI) and Fisher's exact test for binary characteristics (e.g. gender) (Figure 1C). We find that with the exception of two subdivisions, all the remaining 498 subdivisions show a statistically significantly difference in at least one clinical characteristic. This suggests that the shuffling algorithm indeed does identify biologically or clinically distinct divisions of patients in most cases. The frequency with which individual clinical characteristics appear as significantly different between the two groups can therefore be used to identify the combinations of clinical characteristics that co-determine gene expression differences.

Note that the VIStA approach is fundamentally different from clustering techniques like hierarchical or *k*-means clustering. The latter attempt to identify cohesive groups based on similarity, while VIStA, on the contrary, is a divisive algorithm based on maximizing the *differences *between groups. Another important difference to standard clustering approaches is that by design VIStA is able to identify a large number of locally optimal divisions.

#### Technical considerations

We use a relatively low confidence cut-off of FDR≤ 0.1 for the SAM analysis in steps (*i *) and (*ii *) in order to facilitate the emergence of an initial "seed"-grouping. Sensitivity of parameter estimates were robust to variation in the exact choice. Within the SAM framework, the FDR is based on a comparison with random permutations, see [13] for details.

Note that instead of SAM one could also use other approaches to determine the number of differentially expressed genes at each iteration step, for example using the *p-*values of simple t-tests or a minimal fold-change. As VIStA consists of repeated differential expression analyses, the same limitations as for conventional approaches apply for the minimal number of subjects and general data quality.

We implemented a reservoir of 40 subjects (group 3) in order to resemble a gene expression analysis based on extremes, e.g. the 25% of subjects with the lowest BMI vs the 25% of subjects with the highest BMI. In principle, the third group is not strictly necessary, as shuffling can be performed between two groups. Increasing the size of the reservoir group could affect power through selection of more extreme subjects or by reducing the sample size for the differential expression analysis, so it will depend on the concrete application, whether or not a reservoir is useful.

As detailed below, we find that 500 independent runs of VIStA provided sufficient statistical power for a robust distinction between four different subgroups in this study. Generally, a higher number of independent runs could lead to the discovery of more subtle subgroups. It is important to note, however, that the predictive power of the approach is ultimately limited by the quality and size of the expression data, as well as the clinical characteristics.

The algorithm was implemented in the programming language C. A single run with 2,000 iterations takes around three hours on a standard PC. However, the vast majority of the computing time is used to perform the SAM analysis, so using a simpler technique for the differential gene expression analysis would drastically speed up the execution time if necessary.

## Results & discussion

### Differential gene expression of single clinical characteristics

We first attempted to identify statistically significant gene expression differences between patient groups that differ in a single clinical characteristic. To be specific, we aimed to identify genes that were differentially expressed at FDR *<*0.05 using bins of clinical characteristics as presented in Table 2, such as COPD severity, the history of exacerbation or BMI. As shown in Table 2, apart from the severity of airflow limitation as assessed by the GOLD stage, none of the other clinical measures identified significant gene expression changes. This failure suggests that these clinical characteristics are not sufficiently discriminative to capture gene expression variation in COPD. We hypothesized that there are indeed potential molecular drivers to disease heterogeneity, but a single clinical characteristic is unable to capture them. Therefore, we developed an inverse (divisive) clustering methodology to group the 140 COPD patients included in the study based on their gene expression patterns, and then explored the clinical characteristics of the obtained groups (Figure 1).

**Figure 2:**
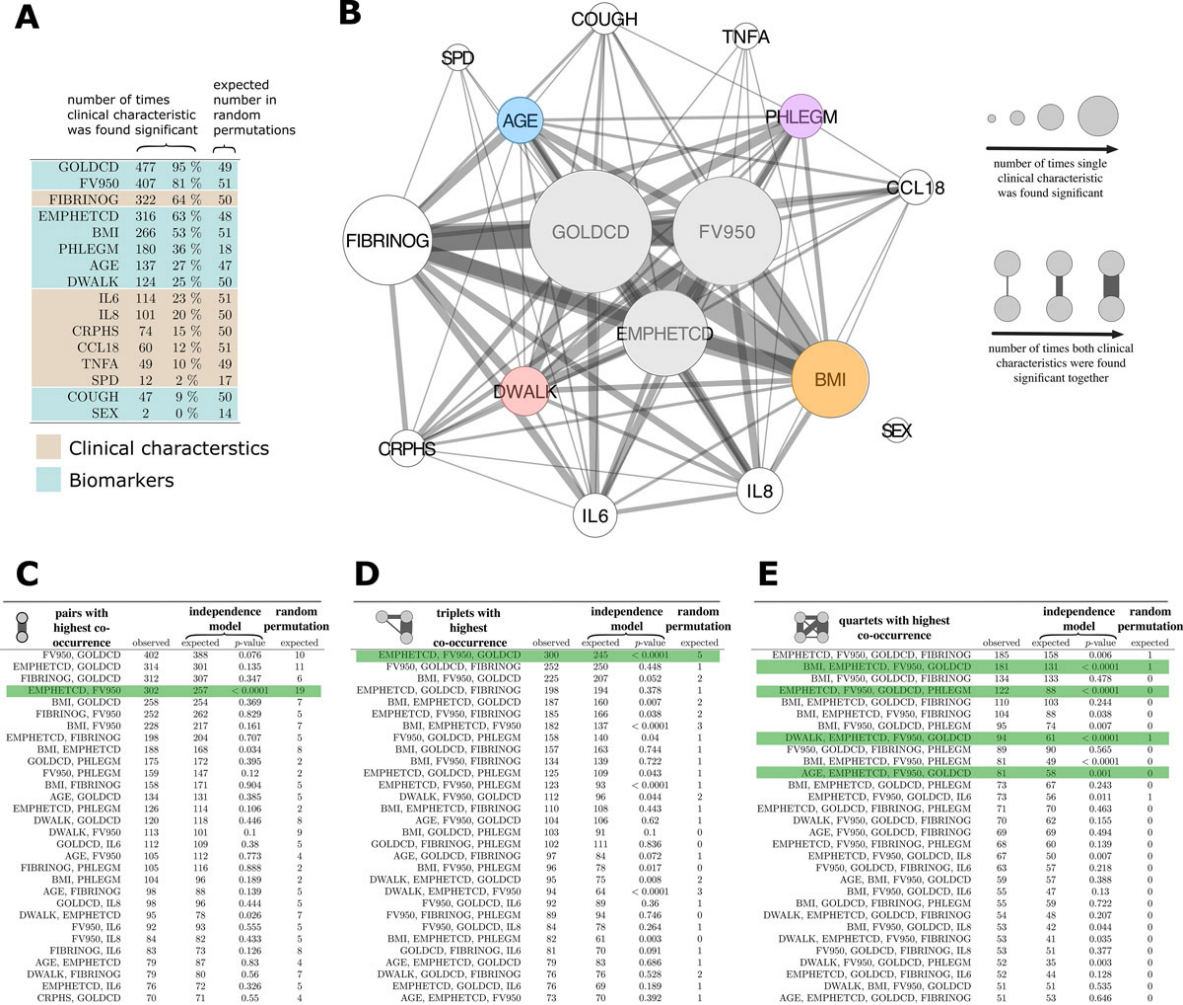
**Combination of clinical characteristics associated with groups from VIStA**. **A **Number of times the characteristics were found significantly different between group 1 & 2 in a total of 500 divisions. Severity of airflow limitation (GOLDCD) is the single most important determinant of differential gene expression, being statistically significant in 95% of all VIStA outputs. **B **Summary of the individual and pairwise number of significant occurrences of the clinical characteristics. Node size is proportional to the number of times a measure was found significant, the width of a link indicates how often two measures appeared significant in the same VIStA division. The core group contains severity of airflow limitation (GOLDCD) and the two emphysema measures EMPHETCD and FV950. **C**, Number of times that pairwise combinations of clinical characteristics co-occurred in the 500 VIStA outcomes. The most significant pair (as compared to a Null model of independent occurrence) is EMPHETCD and FV950, which are both measures of emphysema. **D **The most frequent and significant triplet is a combination of GOLDCD and EMPHETCD and FV950, measuring disease severity. **E **We find significant combinations of the disease severity triplet in B with four clinical characteristics: BMI, PHLEGM, DWALK and AGE.

Figure 2 presents the results of the VIStA analysis, offering a comparison of the clinical characteristics (GOLDCD, FV950, EMPHETCD, BMI, PHLEGM, AGE, DWALK, COUGH and Sex) and inflammatory biomarker levels (interleukin (IL)-6, IL-8, high-sensitivity C-reactive protein (HSCRP), chemokine motif (C-C) ligand 18 (CCL18), surfactant protein D (SPD), fibrinogen (FIBRINOG), and tumor necrosis factor alpha (TNFA) associated with patient subtypes that display the most extreme sputum gene expression pattern differences. We found that the severity of airflow limitation (GOLDCD) was the single most important determinant of differential gene expression, being statistically significant in 95% of all VIStA outputs (*n *= 477, Figure 2A). This is consistent with our finding discussed above that GOLDCD was the only single clinical variable associated with differential gene expression. The second most common clinical determinant of differential sputum gene expression was emphysema, quantified by either density mask analysis (FV950) or assessed qualitatively by the radiologist (EMPHETCD) (81% and 63% of all VIStA outcomes, respectively, Figure 2A) whereas BMI, Phlegm, age and DWALK were observed in 53%, 36%, 27% and 25% of the VIStA outcomes, respectively (Figure 2A). Plasma fibrinogen was the most frequently identified systemic biomarker (64% of all VIStA outcomes),

### Combination of clinical traits from VIStA

Figure 2B illustrates how often combinations (pairs) of significant single clinical characteristics (or inflammatory biomarkers) co-occur in the different VIStA subtypes by the width of the links between them. The statistical significance of each co-occurrence (Figure 2C-E) was calculated using a binomial model that assumes independence of the individual characteristics or biomarker levels as the Null hypothesis. In order to quantify the extent to which the VIStA outcomes could reflect spurious associations, we also generated 10,000 random divisions of the patients and analyzed how often the individual characteristics and their combinations appear as significant (Figure 2C-E). We find that the divisions obtained by VIStA show a much higher number of significant clinical characteristics than expected by chance, with the exceptions of the biomarkers CCL18, TNFA and SPD and the variables COUGH and SEX. Similarly, also combinations of significant characteristics appear more frequent than for randomly assigned division. We observed (Figure 2C) that the pairwise co-occurrences of clinical characteristics and inflammatory biomarkers were dominated by airflow limitation severity (GOLDCD). Other characteristics frequently observed in combinations include emphysema (EMPHETCD or FV950), fibrinogen levels, phlegm, BMI and age. Most pairs appear with the frequency expected for the Null hypothesis of independent individual clinical characteristics (see the many non-significant *p-*values in Figure 2C-E), implying that their association is not significant (e.g. EMPHETCD and GOLDCD). A notable exception is EMPHETCD and FV950, whose statistical association is expected, given that the two variables are not independent but are different measures of the same clinical characteristic (emphysema). Figure 2D, E shows the observed and expected co-occurrence of triplets and quartets of clinical characteristics and inflammatory biomarkers. The most frequent and significant triplet consists of severity of airflow limitation (GOLDCD) and the two emphysema measures EMPHETCD and FV950. GOLDCD and either one of the severity of emphysema measures FV950 or EMPHETCD co-occurred in almost all triplets, which is again expected given their pathobiological relationship in patients with COPD. Figure 2E lists the most frequent combinations of four variables. We find that the most significant combinations are those which include the triple GOLDCD, FV950 and EMPHETCD, together with one additional variable, the most significant being FIBRINOGEN, BMI, PHLEGM, DWALK and age. In the following, however, we have not considered fibrinogen as the basis for a subtype since it is a biomarker rather than a clinical characteristic.

In summary, Figure 2C-E suggests four distinct clinical parameters that define groups of patients with considerable gene expression differences. In all groups the patients are characterized by different disease severity (GOLDCD) and emphysema (i.e. EMPHETCD and FV950) but in addition, each group also has one clear distinctive parameter: high/low BMI (Group I), exercise capacity (DWALK) (Group II), Age (Group III) or presence/absence of phlegm production (Group IV) (Table 3). For example, group IA has high GOLDCD, emphysema, FV950 and low BMI, while group IB has low GOLDCD, emphysema, FV950 and high BMI.

**Table 3:** Summary of the clinical measures, biomarkers, and cell counts among the four groups of COPD patients identified from the results of Figure 2: each group combines GOLDCD, EMPHETCD and FV950, with either BMI (Group I), DWALK (Group II), AGE (Group III) or Phlegm (Group IV).

	**Group-IA** **(n = 25)**	**Group-IB, (n = 23)**	***p-*values**	**Group-IIA** **(n = 21)**	**Group-IIB **, **(n = 32)**	***p-*values**	**Group -IIIA (n = 15)**	**Group-IIIB** **(n = 28)**	***p*-values**	**Group-IVA** **(n = 20)**	**Group-IVB** **(n = 26)**	***p-*values**
	
Age	65.4	65.4	-	63.9	65.4	-	58.73	68.7	***	63	65.96	-
Lung Function			-			-			-			-
FEV1	1.72	0.89	***	1.7	0.9	***	1.72	0.93	**	1.79	0.9	***
FEV1/FVC (%)	57.88	32.43	***	55.0	32.5	***	56.53	33.93	***	57.1	33.04	***
FEV1 reversibility (%)	7.64	4.73	***	11.5	7.2	***	11.62	5.93	***	10.4	5.5	***
Radiologist Emphysema			-			-			-			-
Emphysema severity	1.2	4.2	***	1.3	4.2	***	1.336	3.7	***	1.275	4.2	***
Densitometric Emphysema			-			-			-			-
Emphysema at -950 HU	6.98	33.42	***	6.6	31.7	***	7.71	28.09	***	7.06	33.42	***
Airflow Obstruction			-			-			-			-
GOLD Stage	2	3.3	***	2.0	3.3	***	2	3.2	***	2	3.3	***
Body Mass Index	30.76	21.21	***	27.3	24.2	*	29.87	25.8	-	28.67	24.42	*
Chronic Bronchitis (ATS_CB) 1 = no-CB Phelgm	1 = 24%	1 = 30.4%	-	1 = 85.7%	1 = 62.5%	-	1 = 6.7%	1 = 37%	-	1-100%	1 = 46.2%	***
1 = no chronic phlegm	1 = 56%	1 = 35%	-	1 = 62%	1 = 41%	-	1 = 66.6%	1 = 33%	-	1-100%	1 = 0%	***
6 Minute Walk Distance	428.32	330.02	**	508.8	273.9	***	438.97	321.83	**	462.59	322.9	**
Exacerbations 0 = no-Exacerbations	0 = 68%	0 = 34.8%	-	0 = 71.4%	0 = 28.1%	**	0 = 60%	0 = 37%	-	0 = 70%	0 = 38.5%	*
CCL6	7.3	6.33	-	7.0	6.8	-	6.19	6.73	-	8.64	6.9	-
IL6	5.65	20.2	-	4.3	18.6	-	2.79	6.89	**	3.72	18.53	-
IL8	8.88	10.77	-	8.3	9.4	-	7.5	10.28	*	9.8	10.65	-
TNFa	26.99	160.32	-	31.7	162.9	-	2.35	60.44	-	24.14	142.3	-
CCL18	130.3	117.59	-	126.9	124.4	-	115.8	117.94	-	134	126.19	-
CRPHS	10.4	9.6	-	9.9	9.5	-	5.7	9.72	-	510	8.5	-
FIBRINOG	494.9	499.1	-	481.0	506.2	-	456.8	498.58	-	510.8	489.84	-
SPD	129.64	110.94	-	124.9	119.1	-	79.73	116.3	*	138.76	109.7	-
mMRC	3	2.09	**	1.0	2.4	***	1.21	2.04	*	1.1	2.04	*
SGRQ	43.29	55.55	**	35.9	56.0	***	41.8	52.87	*	36	57.73	***
FFMI	19.53	16.13	***	18.5	17.1	*	18.48	17.8	-	18.83	17.15	*
% Fat (Tissue)	34.92	29.08	**	31.0	31.7	-	35.96	31.94	-	32.69	31.25	-
			-			-			-			-
Neutrophils, % Neut_Blq	61.38	64.87	-	60.7	67.0	**	61.34	66.69	*	62.08	65.55	-
Eosinophils, % Eos blq	3	3.1	-	3.5	3.1	-	2.48	2.92	-	3.26	3.3	-
Lymphocytes, % lymhblq	28.63	24.84	-	28.6	23.2	*	29.19	23.55	*	27.77	23.688	-

* = *p*-value < 0.05; ** = *p*-value < 0.01; *** = *p*-value < 0.0001; - = not significant

To further characterize these subtypes suggested by VIStA we subdivided the full set of all 140 ECLIPSE patients according to the identified clinical characteristics, resulting in 8 groups of 15 to 28 patients. First, we explored a number of clinical, biomarker and cell count measures of the subjects in each group. We find, for example, that serum levels of the biomarkers IL6, IL8 and SPD are significantly higher in group IIIB than in IIIA, a difference that was not observed in other groups. Similarly, the proportion of neutrophils and lymphocytes in sputum were significantly higher in group IIIB in comparison to IIIA (Table 3).

**Figure 3:**
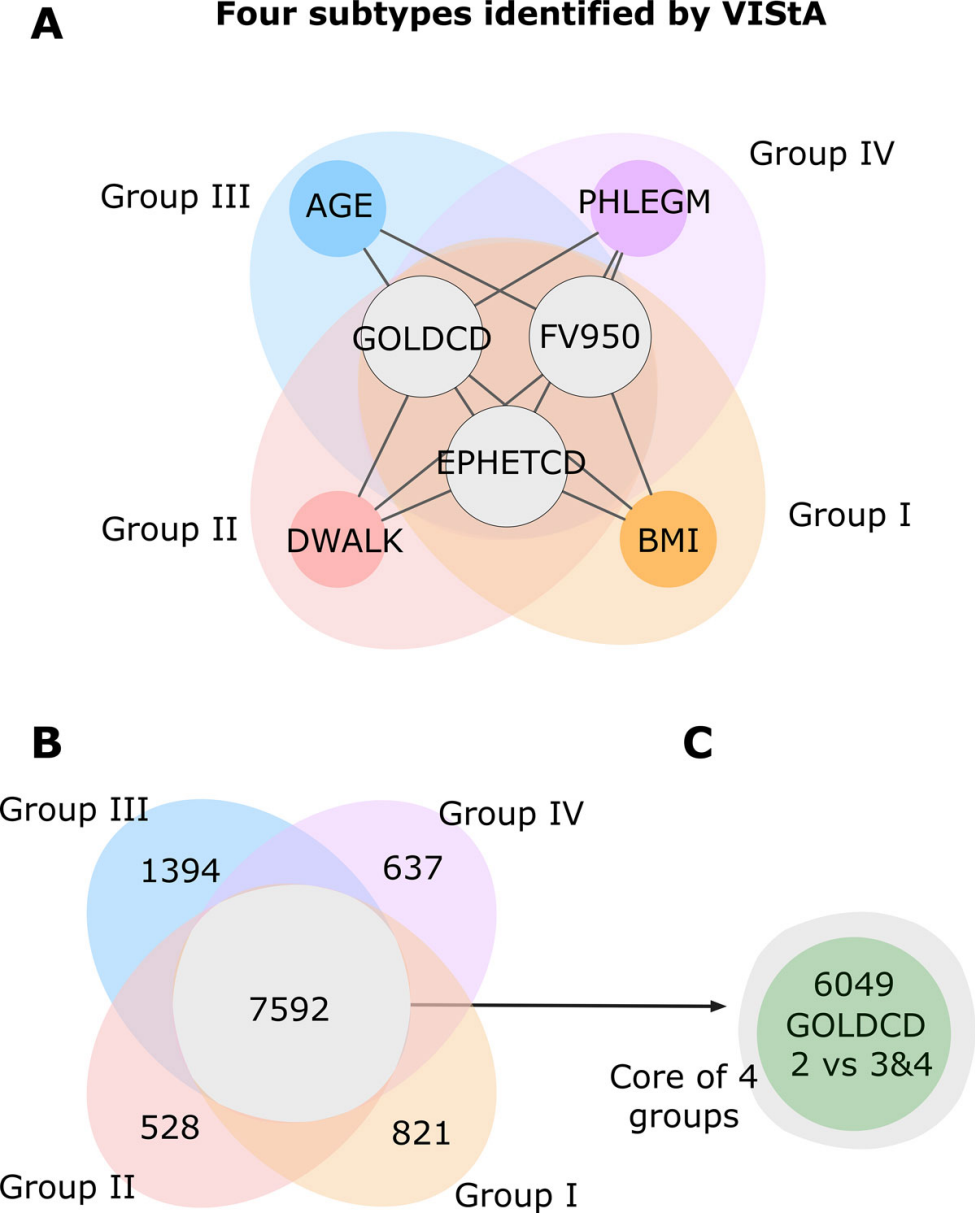
**Four subtypes and differentially expressed genes**. **A **The combinations of phenotypic measures that define the subtypes predicted by the VIStA method: all four subtypes share a common core of high values of GOLDCD, FV950 and EMPHETCD, reflecting disease severity. Each of the individual subtypes I-IV presents one additional clinical characteristic: BMI (subtype I), DWALK (II), AGE (III) or PHLEGM (IV). **B **Venn diagram showing the number of differentially expressed genes unique to each subtype, as well as common to all four subtypes. The common genes show a large overlap with the genes differentially expressed between subjects with GOLDCD 2 and subjects with GOLDCD 3&4, indicating that these genes reflect mostly disease severity.

We then performed a separate differential gene expression analysis (now with a more stringent FDR *<*0.05) on the subgroups, finding 821 unique genes for Group I, 528 for Group II, 1,394 genes for Group III and 637 for Group IV (Figure 3B). The four groups share 7,592 genes that are differentially expressed in all of them. As expected, 80% of these genes were previously identified as differentially expressed comparing patients with moderate (GOLD 2) with those with more severe disease (GOLD 3&4) (Figure 3C). We conclude that the common core is dominated by severity of COPD, while the uniquely differentially expressed genes between the groups represent additional variation.

### Specific genes & pathways in the groups from VIStA

For a further evaluation of the molecular level differences among the four groups, we performed a pathway enrichment analysis for the core set of genes common to all groups, as well as for the unique gene set of each group. Pathway annotations were obtained from the Molecular Signatures Database (MSigDB) published by the Broad Institute, Version 3.1 [14]. MSigDB integrates several different pathway databases, we use KEGG, Biocarta and Reactome. The enrichment analysis between a given gene set and a pathway was done using Fisher's exact test. As shown in Table 4, the top pathways show little overlap between the four groups, providing further evidence for VIStA's ability to capture molecular elements that are specific to each subtype. Several identified pathways were related to metabolism, diabetes and inflammation. Group 1 was most enriched with inflammatory pathways including for example the FC-Gamma-R mediated phagocytosis (*p *= 0.007) and CDC6-association with ORC:origin-complex pathways (*p *= 0.15). Further pathways include small lung cancer (*p *= 0.004) and maturity onset diabetes of the young (*p *= 0.009) [15]. Group II was enriched with lipid transport and beta-cell and insulin signaling pathways like beta cell (*p *= 0.005), HDL mediated lipid transport (*p *= 0.006) and GTP hydrolysis pathways (*p *= 0.007). In group III, pathways related to cell cycle control like mitotic prometaphase (*p *= 0.0048), and downstream signaling pathways (*p *= 0.003) with innate-immunity and GAB1 signaling were enriched. In group IV, distinct gap channel and inflammation pathways were identified like peptide ligand binding (*p *= 0.0006), gap-junction assembly (*p *= 0.0008) and chemokine signaling pathways (*p *= 0.0013).

**Table 4:** The 10 most strongly enriched pathways in the set of genes common among all four groups described in table 3, as well as in the individual gene sets of each group.

Top ten pathways among Common Genes			
**pathway**	***p-value***	**overlap**	**all pathway genes**
REACTOME_GENE_EXPRESSION	*1.22E-35*	235	425
REACTO ME_DIABETES_PATHWAYS	1.91E-33	214	383
REACTOME_METABOLISM_OF_PROTEINS	9.48E-28	134	215
REACTOME_CELL_CYCLE_MITOTIC	7.34E-25	167	306
REACTOME_GLUCOSE_REGULATION_OF_ INSULIN_SECRETION	1.24E-23	104	161
KEGG_HUNTINGTONS_DISEASE	3.16E-23	114	185
REACTOME_INTEGRATION_OF_ENERGY_METABOLISM	1.09E-21	130	229
REACTOME_ELECTRON_TRANSPORT_CHAIN	1.11E-21	60	75
REACTOME_RNA_POLYMERASE_I_III_AND_MITOCHONDRIAL_TRANSCR.PT.ON	2.72E-21	82	120
REACTOME_INFLUENZA_LIFE_CYCLE	1.11E-20	89	137

**Top ten pathways among Group 1 Genes**			

**pathway**	***p*-value**	**overlap**	**all pathway genes**
REACTOME_INORGANIC_CATION_ANION_SLC_TRANSPORTERS	0.00133586	7	94
KEGG_SMALL_CELL_LUNG_CANCER	0.00359651	6	84
KEGG_FC_GAMMA_R_MEDIATED_PHAGOCYTOSIS	0.00723812	6	97
KEGG_MATURITY_ONSET_DIABETES_OF_THE_YOUNG	0.00921957	3	25
REACTOME_AM.NO_ACID_AND_OLIGOPEPTIDE_SLC_TRANSPORTERS	0.00984371	4	48
REACTOME_SLC_MEDIATED_TRANSMEMBRANE_TRANSPORT	0.01009928	8	169
KEGG_B_CELL_RECEPTOR_SIGNALING_PATHWAY	0.01020969	5	75
KEGG_GLYCOSPHINGOLIPID_BIOSYNTHESIS_LACTO_AND_NEOLACTO_SERIES	0.0102887	3	26
REACTOME_NUCLEAR_RECEPTOR_TRANSCRIPTION_PATHWAY	0.01133769	4	50
REACTOME_CDC6_ASSOCIATION_WITH_THE_ORC:ORIGIN_COMPLEX	0.01516009	2	11

**Top ten pathways among Group II Genes**			

**pathway**	***p*-value**	**overlap**	**all pathway genes**
REACTOME_REG ULATION_OF_GENE_EXPRESSIO N_IN_B ETA_CELLS	0.00552	5	101
REACTOME_HDL_MEDIATED_LIPID_TRANSPORT	0.00637	2	11
REACTOME_GTP_HYDROLYSIS_AND_JOINING_OF_THE_60S_RIBOSOMAL_SUBUNIT	0.00675	5	106
REACTOME_FACILITATIVE_NA_INDEPENDENT_GLUCOSE_TRANSPORTERS	0.00759	2	12
REACTOME_REGULATION_OF_BETA_CELLDEVELOPMENT	0.00911	5	114
REACTOME_TRANSLATION	0.01121	5	120
REACTOME_TRANSMEMBRANE_TRANSPORT_OF_SMALL_MOLECULES	0.01148	7	218
REACTOME_IRS_RELATED_EVENTS	0.01182	4	79
REACTOME_INFLUENZA_LIFE_CYCLE	0.01889	5	137
REACTOME_DEADENYLATION_OF_MRNA	0.02469	2	22

**Top ten pathways among Group III Genes**			

**pathway**	***p*-value**	**overlap**	**all pathway genes**
REACTOME_DOWN_STREAM_SIGNAL_TRANSDUCTION	0.00302075	5	35
REACTOME_GAB1_SIGNALOSOME	0.00324484	3	11
REACTOME_SIGNALING_IN_IMMUNE_SYSTEM	0.00470548	20	366
REACTOME_MITOTIC_PROMETAPHASE	0.00489389	8	92
REACTOME_INNATE_IMMUNITY_SIGNALING	0.00584887	10	136
REACTOME_SIGNALLING_TO_RAS	0.0060289	4	26
REACTOME_FORMATION_OF_PLATELET_ PLUG	0.00753191	12	186
REACTOME_GRB2_SOS_PROVIDES_LINKAGE_TO_MAPK_SIGNALING_FOR_INTERGRINS	0.00821656	3	15
REACTOME_MYOGENESSIS	0.00895252	4	29
REACTOME_HEMOSTASIS	0.01301061	15	274

**Top ten pathways among Group IV Genes**			

**pathway**	***p*-value**	**overlap**	**all pathway genes**
REACTOME_PEPTIDE_LIGAND_BINDING_RECEPTORS	0.00059	12	173
REACTOME_GAP_JUNCTION_ASSEMBLY	0.00076	4	19
KEGG_CHEMOKINE_SIGNALING_PATHWAY	0.00133	12	190
REACTOME_GAP_JUNCTION_TRAFICKING	0.00340	4	28
REACTOME_CHEMOKINE_RECEPTORS_BIND_CHEMOKINES	0.00787	5	55
REACTOME_ACTIVATION_OF_ATR_IN_RESPONSE_TO_REPLICATION_STRESS	0.00936	4	37
REACTOME_SIGNALING_IN_IMMUNE_SYSTEM	0.00943	16	366
KEGG_T_CELL_RECEPTOR_SIGNALING_PATHWAY	0.01126	7	108
REACTOME_CELL_CYCLE_CHECKPOINTS	0.01237	7	110
KEGG_NATURAL_KILLER_CELL_MEDIATED_CYTOTOXICITY	0.01263	8	137

Finally, we identified genes with at least a 2-fold change (FC) in expression [16,17] at an FDR of *<*0.05, see Table 5 for the specific set of upregulated and downregulated genes in each subgroup. For example, *MMP7 *was found to be upregulated in group I (BMI), consistant with findings in [18], where nutritionally induced obese mice showed alterations in *MMPs *and *TIMPs *expression, thus providing further evidence for the role of these proteolytic system genes in COPD subtype with low BMI.

**Table 5:** Top ten upregulated and downregulated unique genes and their fold-change (FC) in each group (In group II, only five unique genes are downregulated).

Group 1		Group II		Group III		Group IV	
**Gene**	**FC**	**Gene**	**FC**	**Gene**	**FC**	**Gene**	**FC**

LOC100127940	2.8	RP-3377H14.5	2.4	DDX3Y	4.6	IL1F9	2.5
PDCD6	2.4	ZFYVE16	2.2	EIF1AY	3.2	IL23A	2.5
AHRR	2.4	TGFBR1	2.2	HELB	3	TUB	2.4
CD1B	2.4	MARCH6	2.2	LOC100130224	2.9	GJB2	2.3
KIT	2.4	CAPZA1	2.2	UTY	2.9	CD22	2.3
CADM1	2.3	KIAA0319	2.2	ADORA3	2.9	FAF1	2.3
MMP7	2.3	DHX36	2.2	ARNT2	2.9	MB0AT7	2.3
C20orf197	2.3	DLGAP4	2.1	CXCL14	2.6	SULT2A1	2.3
RNF144A	2.2	RIF1	2.1	TMEM61	2.6	TMEM88	2.3
MYO1B	2.2	NT5C2	2.1	PPARGC1B	2.6	CHST7	2.3

SGK493	-2.0	TIFAB	-2.0	C1orf201	-2.5	VASH1	-2.3
ALS2CR4	-2.1	CCDC42	-2.2	ST3GAL3	-2.5	LINC00607	-2.3
ENPP5	-2.2	HBE1	-2.2	APOOL	-2.6	KLHDC7B	-2.3
FLJ14082	-2.2	NAPSB	-2.2	IL28RA	-2.6	DHODH	-2.3
LOC1441204	-2.2	C4orf7	-3.8	ZNF624	-2.6	CDDC113	-2.3
L0C100134569	-2.2			SMAD5	-2.6	IGF2BP3	-2.3
FAM101A	-2.7			NRP1	-2.6	C3orf27	-2.3
LOC92270	-2.8			LOC654342	-2.6	ZNF618	-2.3
HPR	-2.9			TSIX	-3.3	AKR1C4	-2.4
HP	-2.9			XIST	-4.1	LOC401321	-2.4

## Conclusion

We have found that with the exception of severity of airflow limitation, categorizing COPD subtypes according to a single clinical characteristic does not yield groups of patients with significant gene expression differences. In this study, we therefore introduced a novel methodology that allowed us to identify *combinations *of clinical characteristics that correspond to clear gene expression differences.

Our results suggest that while gene expression differences are mainly driven by the severity of airflow limitation and the extent of emphysema, a smaller, yet discriminative contribution is also observed for a set of additional clinical characteristics: BMI, distance walked, age and chronic phlegm production, each defining a subtype of patients. Validation of these groups and the underlying pathways will require replication in a second cohort of COPD subjects. Note that additional differences may also exist for clinical characteristics that have not been considered in the present study.

The observed subgroups with combinations of different clinical characteristics are consistent with the clinical heterogeneity of COPD, where a given patient may manifest more than one measurable feature of COPD, suggesting either that the underlying mechanisms contribute to more than one feature or that multiple mechanisms are maladapted in an individual.

While we focused on COPD in this study, the proposed VIStA method can be more generally applied to any other complex, heterogeneous disease and presents a promising approach to the important problem of disease heterogeneity and subtyping/subgrouping. A better understanding of this problem is invaluable, for example, for improving the selection of patients for evaluating novel agents. To the extent that gene expression reflects genetic and epigenetic variation, the subtypes identified by our method may further suggest different approaches to identifying genetic susceptibility.

## Competing interests

RJM, RTS, BEM, NL. JR, CL, GB are employees of GlaxoSmithKline and own shares and share options in the company.

## Authors' contributions

JM, AS, ALB carried out the analysis and wrote the manuscript; AA, BC, SR, ES, RTS, BM, JR, NL provided expertise on the ECLIPSE data and COPD and wrote the manuscript; MHC, RM, SG, CL, GB advised on the analysis and wrote the manuscript.

## Declarations

The publication costs for this article were funded by Northeastern University, MA, USA.

This article has been published as part of *BMC Systems Biology *Volume 8 Supplement 2, 2014: Selected articles from the High-Throughput Omics and Data Integration Workshop. The full contents of the supplement are available online at http://www.biomedcentral.com/bmcsystbiol/supplements/8/S2.
